# Combination Insecticide Treatments with Methoprene and Pyrethrin for Control of Khapra Beetle Larvae on Different Commodities

**DOI:** 10.3390/insects15010077

**Published:** 2024-01-22

**Authors:** Deanna S. Scheff, Frank H. Arthur, Michael J. Domingue, Scott W. Myers

**Affiliations:** 1 Center for Grain and Animal Health Research, Agricultural Research Service, USDA, 1515 College Avenue, Manhattan, KS 66502, USA; fha@ksu.edu; 2 Forest Pest Methods Laboratory, Plant Protection and Quarantine, Animal and Plant Health Inspection Services, USDA, 1398 West Truck Road, Buzzards Bay, MA 02542, USA; michael.j.domingue@usda.gov (M.J.D.); scott.w.myers@usda.gov (S.W.M.)

**Keywords:** grain protectant, khapra beetle, residual protection, stored products, methoprene, deltamethrin, development index

## Abstract

**Simple Summary:**

The khapra beetle is considered by many to be one of the most destructive stored product pests in the world and it is a quarantine species of significant concern in the United States. This beetle is known to feed on >100 commodities, leaving behind frass, broken and damaged grain kernels, and cast skins, which can make products unsuitable for consumption. Because its immature stage is so problematic, it is critical to identify effective management strategies, which could include fumigation, trapping, and insecticides. In this study, we investigated whether two different grain protectants applied to bulk corn, wheat, and brown rice could be efficacious for inhibiting the development from larvae to adult. Both grain protectants reduced adult emergence on brown rice for 6 months of storage and wheat and corn for up to 12 months of storage. Additional studies are needed to directly measure the amount of frass, feeding damage, and/or insect-damaged kernels (IDK), and larval weight, which would provide further insight on the relationship between commodity type and the efficacy of the insecticide application. Reducing adult emergence inhibits reproduction and population growth during storage. Thus, grain protectants can be a useful preventative tactic for stored commodity managers against the khapra beetle.

**Abstract:**

*Trogoderma granarium* Everts, the khapra beetle, is a serious pest of stored products throughout the world. Larvae pose a significant threat to stored products because they feed on >100 different commodities, possess the ability to enter facultative diapause, and are difficult to detect. Control methods for *T. granarium* include fumigation, contact insecticides, trapping, and insecticide-incorporated packaging. The objective of this study was to determine the residual efficacy of two insecticide formulations (methoprene + deltamethrin + piperonyl butoxide synergist Gravista^®^ and methoprene + deltamethrin, DiaconIGR^®^ *Plus*). These insecticides were evaluated on three stored product commodities, corn, wheat, and brown rice, by exposing *T. granarium* larvae during a 12-month testing period. Both formulations significantly reduced adult emergence on corn and wheat for 12 months and on brown rice for up to 6 months. Adult emergence was highest at month 12 for corn (8.41%), and brown rice (85.88%), and month 9 for wheat (39.52%), treated with DiaconIGR^®^ *Plus* or Gravista^®^, respectively. A biological index used to measure the development of exposed larvae on the treated grain from the larval stage (low values) to adult emergence (high values) was lower (fewer adults) on corn and wheat compared to controls. Despite differences in formulations, each of these grain protectants could be utilized by stored commodity managers to protect commodities during storage and transportation for *T. granarium* when and if this pest is detected at USA ports of entry.

## 1. Introduction

*Trogoderma granarium* Everts, the khapra beetle, is a serious pest of stored products worldwide. *Trogoderma granarium* larvae are known to feed on whole and broken kernels of >100 different commodities and larvae can enter a facultative diapause under less-than-ideal conditions such as low temperature, low humidity, crowded conditions, and lack of food resources [1,2,3,4,5,6]. As a result, diapause can continue for months or years until favorable conditions return. This makes it difficult to detect low-level infestations in stored bulk grain, food processing and storage facilities, and packaged products. The eradication of established populations is also challenging, as *T. granarium* often resides in cracks and small spaces and can readily move to new food resources or re-infest commodities after control efforts [7,8]. Thus, an integrated pest management (IPM) program that focuses on long-term prevention and control of this species using multiple surveillance and control methods is critical. An efficient IPM program utilizes all available monitoring and control options to manage the stored products, and does not rely on a single control tactic. One type of control tactic is the use of grain protectants on stored commodities to prevent infestation on stored products during extended storage periods.

Grain protectants are insecticides that are applied to raw, uninfested grains that are loaded into storage to provide residual control of the grain during the entire time the grains are stored [9]. Most grain protectants in the United States of America (USA) are applied to the entire grain mass as they are unloaded into a bin or silo with the goal of coating as many kernels as possible prior to long-term storage on farms, agricultural cooperatives, or commercial grain storage facilities. Unlike fumigants, which have no residual efficacy, grain protectants can be effective for weeks to months after application. Additionally, registered grain protectants could be used by exporting countries to protect commodities during storage and transportation to prevent costly re-exportation or destruction of *T. granarium*, or other stored product insect-infested commodities upon reaching an importing country.

Grain protectants often utilize insect growth regulators (IGRs) to control the juvenile stages of the insect species by inhibiting molting and development to the adult stage [10]. One such grain protectant is methoprene (DiaconIGR^®^, Central Life Sciences, Schaumberg, IL, USA). This product contains ~300 mg/mL active ingredient (a.i.) methoprene and is applied for labels ranging from 105 to 60 mL (per 1000 bushel) depending on the stored commodity. Methoprene has low mammalian toxicity, and when used at label rates, there is little to no effect on non-target organisms [11]. Previous research has found that wheat and brown rice treated with 1.25 or 2.5 ppm (mg/kg) methoprene inhibited the progeny production of *Rhyzopertha dominica* (F.), lesser grain borer, for up to 24 months [12]. Additionally, methoprene-treated wheat and corn suppressed the progeny production of *Tribolium castaneum* (Herbst), red flour beetle, in the same 24-month study [12].

However, IGRs generally do not affect adult beetles and must be combined with a contact insecticide to target adults in addition to the juvenile stages. Previous research investigating the combined effect of methoprene and deltamethrin on controlling *R. dominica, Sitophilus oryzae* (L.), rice weevil, and *T. castaneum* adults on brown rice and maize found that one combined formulation (1.0 ppm deltamethrin and 2.5 ppm methoprene) on brown rice inhibited all progeny production of *R. dominica* and the total weight loss was <0.01% [13]. The same formulation also resulted in a ~97% reduction in *S. oryzae* progeny produced from exposed parental adults and a significant reduction in total weight loss, indicating there is a possible additive effect of the deltamethrin and methoprene combination [13].

Recently this combination of methoprene and deltamethrin has been further advanced with the incorporation of the synergist piperonyl butoxide (PBO) into the formulation. As a synergist, PBO aids in preventing the enzymes in the insect body from activating and increasing pyrethrin/pyrethroid effectiveness [14,15]. With the addition of PBO to insecticide formulations, the amount of pyrethrin/pyrethroid added to the insecticide formulations can be reduced and remain effective [14]. Thus, the objective of this study was to determine the residual efficacy of the new insecticide formulation (methoprene + deltamethrin + PBO, Gravista^®^) compared to the standard formulation (methoprene + deltamethrin, DiaconIGR^®^ *Plus*) applied to three different commodities over 12 months of simulated storage.

## 2. Materials and Methods

### 2.1. Insecticide Formulations

There were two insecticide formulations used in this study. The first was a commercially available deltamethrin + methoprene combination (DiaconIGR^®^ *Plus*, Central Life Sciences, Schamburg, IL, USA), and henceforth referred to by the trade name. This formulation contained 4.75% a.i. deltamethrin, 11.40% a.i. *s*-methoprene, 83.85% other ingredients, alternatively expressed as 49 mg a.i./mL of deltamethrin and 120 mg a.i./mL *s*-methoprene.

The second insecticide formulation used in this study is a new formulation containing lower amounts of methoprene and deltamethrin but including PBO, with the given trade name of Gravista^®^, and henceforth referred to by the trade name. The new insecticide formulation was provided by an industry cooperator, Central Life Sciences, as part of a cooperative research program. The formulation contained 1.20% a.i. deltamethrin, 2.85% a.i *s*-methoprene, and 33.30% a.i PBO or 12.0 mg a.i/mL deltamethrin, 27.4 mg a.i/mL *s-*methoprene, and 320.0 mg a.i/mL PBO. The insecticide formulation provided by the industry cooperator was a laboratory formulation before full registration with the U.S. Environmental Protection Agency (EPA), and thus label rates used in this study were “proposed label rates”. Gravista^®^ has since been registered for use (EPA. Reg. No. 8459-116), and the label rates differ slightly from those used in this study.

### 2.2. Commodity Treatments

#### 2.2.1. Wheat

According to the insecticide label, DiaconIGR^®^ *Plus* is to be applied at a rate of 566 mL in 18.9 L of water to treat 1000 bsh of wheat. The weight approximation of wheat is 27.22 kg/bsh, which requires 0.7 mL of insecticide mixed with 25 mL of water; 0.4 mL of the insecticide solution was then applied to 500 g of wheat using an artist spray brush (Model 105, Bader Company, Franklin Park, IL, USA). The proposed label rate for Gravista^®^ was 1138 mL per 18.9 L of water to treat 27.27 kg/bsh of wheat. To treat with this rate, 1.5 mL of Gravista^®^ was mixed with 25 mL of water, and 0.4 mL of insecticide was applied to 500 g of wheat using the artist spray bush described above.

#### 2.2.2. Brown Rice

The label rate for brown rice is 424 mL DiaconIGR^®^ *Plus* in 18.9 L of water to treat 1000 bsh of brown rice. Using a weight approximation of 20.45 kg/bsh, 0.5 mL of DiaconIGR^®^ *Plus* was mixed with 25 mL of water; then, 0.5 mL of the solution was applied to 500 g of brown rice using the artist spray brush. Similarly, to treat the brown rice in proportion to the proposed label rate for Gravista^®^, 1.1 mL of formulated product was mixed with 25 mL of water, and 0.4 mL of insecticide was applied to 500 g of brown rice.

#### 2.2.3. Corn

The label rate for corn is 532 mL of DiaconIGR^®^ *Plus* in 18.9 L of water to treat 1000 bsh. Using a weight approximation of 25.45 kg/bsh, 0.7 mL of DiaconIGR^®^ *Plus* was mixed with 25 mL of water and 0.5 mL of solution was applied to 500 g of corn using an artist spray brush. Similarly, the proposed label rate for Gravista^®^ was 1062 mL per 18.9 L of water. To treat the corn, 1.4 mL of Gravista^®^ was mixed with 25 mL of water and 0.4 mL of the insecticide solution was applied to 500 g of corn.

### 2.3. Insects

This study was conducted at the United States Department of Agriculture–Animal and Plant Health Inspection Service, Forest Pest Methods Laboratory (FPML), in Buzzards Bay, MA, USA. *Trogoderma granarium* larvae originated from a 2011 collection in Pakistan and were maintained in the FPML insect containment facility. The beetles were reared on a combination diet of 160 g ground dog food (Purina Dog Chow Complete, Nestlé Purina PetCare Company, St. Louis, MO, USA) mixed with 20 g of wheat germ (The Mennel Milling Company, Fostoria, OH, USA), and sprinkled with 20 g of rolled oats (Heartland Mill, Marienthal, KS, USA) on the surface in a 0.95 L glass jar. The colonies were maintained in an environmentally controlled chamber at 30 °C in complete darkness. The larvae used in this study were classified based on their size (mm) because they have an indeterminant number of molts. Thus, the larvae used in all bioassays were standardized to 3.5–5 mm, which are medium-sized larvae for this species.

### 2.4. Commodity Treatment and Bioassays

Each commodity (wheat, brown rice, and corn) was treated in 15 lots of 500 g samples. Five lots were for DiaconIGR^®^ *Plus* treatments, five lots were for Gravista^®^ treatments, and five lots were for the controls. The individual lots of each commodity were placed in a single layer on the bottom of a plastic bin, 44.5 × 36.2 × 17.8 cm (Sterilite Corp., Townsend, MA, USA). The insecticides were applied according to label rates, and water was applied to the control group. Five replicate 25 mL vials of insecticide were prepared for each treatment and commodity; one vial was used per lot, and thus represented five replications per insecticide and commodity combination. After each commodity lot was treated, it was mixed by gently shaking the grain in the plastic bin for 30 s, and allowed to dry at ambient conditions for 24 hr. After 24 hr, the grain was placed into a 710 mL plastic container and held at ambient conditions for the duration of testing. The testing periods were at month 0 (1 d after treatment), 3, 6, 9, and 12 months of storage at ambient conditions.

At the start of each testing period, an 80 g sample was taken from each lot and placed inside a 0.18 L plastic vial and fitted with a cap and screened lid. Twenty *T. granarium* larvae, 3.5–5 mm in size, were added to each vial and placed inside an environmental chamber at 30 °C in complete darkness for 6 wks (same conditions as insect colonies). The vials were immediately frozen, at −18 °C, at the termination date for two weeks before vials were examined either at the USDA-APHIS or USDA-ARS facility due to quarantine regulations. Therefore, the effect on *T. granarium* was assessed based on the level of development after each testing period. The number of adults, deformed pupae/adult intermediates, normal pupae, and larvae were counted for each treatment.

**Figure 1:**
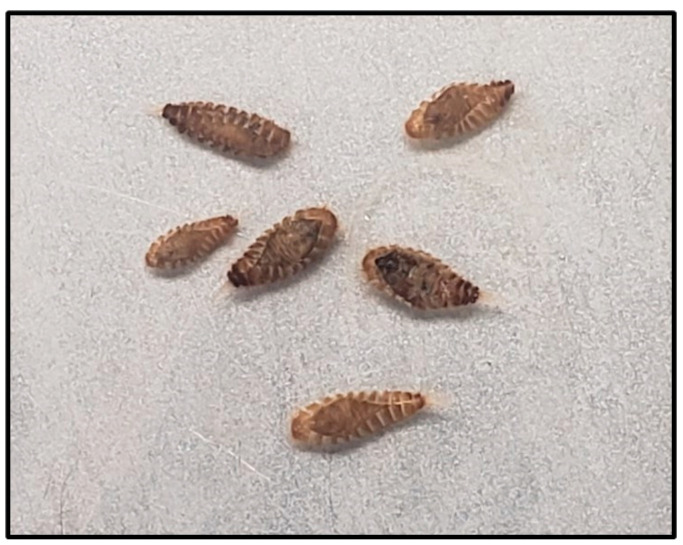
Deformed pupal–adult intermediate stages for *Trogoderma granarium* after exposure to either DiaconIGR^®^ *Plus* or Gravista^®^ applied to corn, wheat, or brown rice and observed after 6 wks of exposure.

A development index was created by grading each life stage (adults, deformed pupae/adult intermediates, normal pupae, and larvae) as follows: individuals remaining in the larval stage were classified as 1, normal pupae were scored as 2, pupal–adult intermediates were scored as 3 (Figure 1), and normal adults were scored as 4. Although 20 individual larvae were placed in each vial for the monthly bioassays, not all larvae were recovered from each vial. Therefore, the number of each life stage recovered was converted to a percentage of 20, and thus the value for the developmental index ranged from 20 (all larvae remaining in that stage) to 80 (all larvae emerging as normal adults).

### 2.5. Data Analysis

The data were analyzed using the Mixed Procedure in the Statistical Analysis System (SAS Institute, version 9.2, Cary, NC, USA) to determine the significance of the main effects of treatment (controls, DiaconIGR^®^ *Plus* and Gravista^®^), commodity (wheat, brown rice, corn), and the bioassay months (0, 3, 6, 9, and 12), for each of the four life stages, and by using the developmental index values. The data were further analyzed for each of the above by month using the Mixed Procedure to determine the significance of treatment and commodity for each life stage and developmental index, and the means were separated using LS Means with Tukey Adjustment (*p* < 0.05).

## 3. Results

### 3.1. Larval Stage

The main effects of treatment, month, commodity, and all associated interactions were significant at *p* < 0.01 for the percentage of *T. granarium* larvae remaining in that stage at the end of the 6 wks observation period (Table 1). It should be noted that the vials were frozen after 6 wks and thus live vs. dead larvae could not be determined. There were significantly more larvae in the treated corn and wheat over all the testing periods compared to the controls (Table 2). In contrast, there were few significant differences in the percentage of larvae in the control and treated brown rice vials over the duration of the experiment (Table 2). At month 6, the percentage of individuals remaining in the larval stage on controls was >60%, which was greater than that on any other month of testing. This same trend was observed in both insecticide treatments.

The data in Table 2 were then analyzed by month to determine differences between the treatment and commodity for individuals remaining in the larval stage at the end of the observation period. The percentage of larvae exposed on corn treated with DiaconIGR^®^ *Plus* and remaining in that stage was >75% throughout the study and was significantly greater than the percentage of larvae exposed on the corn treated with Gravista^®^ throughout the study. The percentage of larvae remaining in that stage on wheat treated with DiaconIGR^®^ *Plus* and Gravista^®^ was lower than the corresponding percentages on corn for all months. The percentages of larvae remaining in that stage after exposure on brown rice treated with either DiaconIGR^®^ *Plus* or Gravista^®^ ranged from 3.1 to 16.7% and was lower than the corresponding percentages on the treated corn, and occasionally lower than the corresponding percentages of individuals remaining in the larval stage on wheat.

### 3.2. Pupal Stage

The main effects of treatment and month, the treatment × month interaction, and the three-way interaction were all significant at *p* < 0.012 or less, but neither the main effect of commodity nor any other interactions were significant for exposed larvae that advanced to the pupal stage (Table 3). The percentage of individuals classified as normal pupae was 5.3% or less except for exposures on wheat and brown rice at two months (Table 4), indicating that most of the individuals that advanced beyond the larval stage also advanced beyond the pupal stage during the observation period. There were few significant differences between commodity or between the DiaconIGR^®^ *Plus* and the Gravista^®^ treatment versus controls (Table 4).

### 3.3. Pupal-Adult Intermediate Stage

The overall ANOVA for pupal–adult intermediates was significant at *p* < 0.01 for all main effects and all interactions (Table 5). Pupal-adult intermediates were rare in the controls, <1% (Table 6). However, there were significantly more pupal–adult intermediates in commodities treated with DiaconIGR^®^ *Plus* and Gravista^®^. In general, the percentages of pupal–adult intermediates were greater in wheat compared to either corn or brown rice, with no differences between the DiaconIGR^®^ *Plus* and Gravista^®^ treatments on wheat except for month 12, when the percentages were higher in the DiaconIGR^®^ *Plus* treatment (Table 6). The lower percentages of pupal–adult intermediates on corn were largely due to the larger percentage of larvae remaining in that stage on corn relative to wheat or brown rice.

### 3.4. Adult Stage

All main effects and interactions were significant for adult emergence at *p* < 0.001 (Table 7). Except for month 6 on all commodities, and month 12 on brown rice, the percentage of adults in controls was above 70% (Table 8). The percentage of adults on corn treated with either DiaconIGR^®^ *Plus* or Gravista^®^ was significantly less than the control with <10% until month 12 when the percentage of those adults on the Gravista^®^ treatment increased to 41% (Table 1). The percentage of adults on the DiaconIGR^®^ *Plus* treated corn was less than corn treated with Gravista^®^. The percentage of adults on the two wheat insecticide treatments was <6% for month 0 to 6. There was a sharp increase in the percentage of adults at months 9 and 12, where the percentage of adults ranged from 37 to 52%. However, the increase in the percentage of adults was still significantly lower than the control. The percentage of adults on the two insecticide treatments on brown rice ranged from 43 to 89%, indicating a lack of efficacy on brown rice compared to corn and wheat (Table 8).

### 3.5. Developmental Index

The final analysis was on the development index, which ranged from 20 (all larvae remaining in that stage) to 80 (all larvae emerging as adults). The overall ANOVA was significant for all main effects and all interactions except for the three-way interaction (Table 9). The development index values were significantly lower on corn treated with DiaconIGR^®^ *Plus* and Gravista^®^ compared to the control. The developmental index was generally lower on treated wheat compared to the control, but overall, no significant differences were observed after month 3. There were no differences in developmental index values between the control and treatments for brown rice. Index values were generally lowest on corn compared to wheat and brown rice, with the greatest index values consistently on rice compared to corn and wheat. There were few differences in index values between DiaconIGR^®^ *Plus* and Gravista^®^ on any commodity (Table 10).

## 4. Discussion

*Trogoderma granarium* is known to feed on multiple commodities; however, the developmental time is commodity-specific [16,17]. In one study [16], newly hatched *T. granarium* larvae were monitored as they fed on barley, rice (*Oryza sativa* L.), rye, wheat, and walnuts. Researchers found the longest developmental time of larvae and pupae to be on walnuts and rice, 91 and 82 d, respectively, and the shortest time was wheat and rye at 54 d [16]. Throughout the 12-month study, we observed significantly more larvae on corn compared to wheat or brown rice treated with DiaconIGR^®^ *Plus* and Gravista^®^. Previous studies on the population growth of *T. granarium* after 60 d on ten common grains (starting from larvae) were conducted and researchers observed the highest population growth was on wheat and triticale and the lowest on peeled barley and maize [17]. Thus, the high number of larvae found in corn after each exposure period could be partly due to its slower developmental time. No dead larvae were observed in the vials with corn after the conclusion of each month. In previous studies investigating insecticides and *T. granarium* larvae, dead larvae appeared black and shriveled. In this study, all larvae appeared to be healthy after the testing period. This suggested that the effect on *T. granarium* larvae development would be a function of the insecticide formulation or slower developmental time. Similarly, the number of larvae in the corn control was also higher than in the other two. Significantly more larvae were observed in treatments of brown rice at month 0 and in wheat up to month 3. As will be discussed below, those larvae continued to develop into pupae or adults, and sometimes in between.

There were few normal pupae observed in the treated or control vials during the study. In the control vials, the larvae developed through the pupal stage to adulthood. However, pupal–adult intermediates or deformities were observed among all commodities and both insecticide treatments (Figure 1). Wheat had a higher percentage of deformed pupae compared with corn and brown rice. The faster development of *T. granarium* on wheat may account for the higher numbers of deformed pupae. The deformities can be attributed to the IGRs in the formulation, which have been documented in other studies with IGRs applied to grain, surfaces, and packaging. Previous experiments with IGRs applied to different surfaces resulted in deformed adult genitalia, incomplete sclerotization of adult legs and antennae, incomplete hardening of elytra, and incomplete larval–pupae or pupae–adult transformations [18,19,20,21]. The deformities are a direct result of the IGR, whereby the insect is arrested in the larval stage, mortality occurs in the intermediate stage, or the result is deformed adults [10].

Both insecticide treatments were highly effective at inhibiting the larvae from reaching the adult stage on wheat and corn for up to 12 months. However, after 6 months of brown rice storage, there was no significant difference between the control and the treated grain samples. Thus, if larvae are not feeding on the brown rice at the same rate as wheat, the effect of the insecticide on the larvae, because of ingestion, would be limited, and more adult beetles would be observed as is the case for brown rice. What may be more revealing of the effect of each treatment is the developmental index for *T. granarium* on each treated commodity. Corn treated with DiaconIGR^®^ *Plus* and Gravista^®^ had the lowest developmental index compared to the control and other treated commodities. The range in indices was 24–31 for DiaconIGR^®^ *Plus* and 27–51 for Gravista^®^, but the ranges were never statistically different between the two treatments. These indices demonstrate that the majority of the larvae exposed to the treated corn remained in the larval stage and began to progress to the pupal and adult stages as the residual time increased. On the other hand, the development index for larvae exposed to DiaconIGR^®^ *Plus* and Gravista^®^ treated with brown rice ranged from 52 to 74 and 47 to 75, respectively, and was not statistically different from the control. This range in index values corresponds to deformed pupal–adults and adult beetles making up the majority of the insect life stages present. This result suggests that DiaconIGR^®^ *Plus* and Gravista^®^ have little effect on *T. granarium* development when applied to brown rice.

Deltamethrin, a pyrethroid, and methoprene, an IGR, affect insects through ingestion and/or direct contact with the insect. IGRs affect the growth and development, and pyrethroids affects the insect’s nervous system, and both will cause mortality upon extended exposures. Previous studies on the use of pyrethroids and IGRs applied as contact insecticides to various surfaces have demonstrated immediate and residual efficacy on *T. granarium* larvae and adults [7,22,23,24,25,26]. In these studies, adult *T. granarium* are generally more susceptible than larvae to several insecticides applied to grain or concrete surfaces [24,26]. Adult *T. granarium* are short lived [6,26] and exposure to treated surface or grain protectants would be short compared to the larval stage. Deltamethrin and cyfluthrin pyrethroid insecticide formulations were found to be more effective on *T. granarium* larvae compared to the IGRs methoprene and pyriproxyfen, with reduced adult emergence and greater larval mortality when exposed to five difference surfaces [7]. It has been stated that *T. granarium* larvae are affected by direct contact with the insecticide; however, the result may not be death and recovery may occur [22]. The body of *T. granarium* is covered with long short hairs [27]. It is theorized that the larval hairs on the insect’s body create a semi-barrier to the treated surface, whether that is grain kernel or a surface, and inhibit direct contact with the insecticide.

The effectiveness of the insecticides used in this study relies on two factors: (1) the food substrate being directly treated and consumed (ingestion); and (2) the absorption from movement on the treated grain (contact). Thus, if minimal feeding occurs based on the type of commodity, i.e., rice and corn are less than wheat [16], then it is predicted there will be a reduction in insecticidal efficacy because there is a greater reliance on contact efficacy. In contrast, if there is more feeding, it is likely that either incomplete metamorphosis or death will occur based on the dual insecticide modes of action in the formulations. As observed in this study and prior research, there are many factors contributing to *T. granarium* growth and development, thus making it difficult to tease out one specific contributing factor. Additional studies are needed to directly measure the amount of frass, feeding damage, and/or insect-damaged kernels (IDK), and larval weight, which would provide further insight into the relationship between commodity type and the efficacy of the insecticide application and primary route of insecticide entry. In addition, future surveillance and monitoring is needed to monitor for potential pesticide resistance to the active ingredients in the formulations studied.

Overall, based on the results of the effect of DiaconIGR^®^ *Plus* and Gravista^®^ on *T. granarium* larvae and the resulting development index, these treatments were most effective on corn and wheat and minimally effective on brown rice. The new Gravista^®^ product, with ~75% less a.i. deltamethrin and methoprene in the formulation and the addition of PBO, performed similarly to the standard DiaconIGR^®^ *Plus* product for stored grain protection. Follow-up studies are needed to evaluate additional commodities, longer storage or exposure periods, and different temperature and humidity conditions. In addition, investigating the effects of grain protectants on diapausing *T. granarium* is also warranted.

## Figures and Tables

**Table 1:** Three-way ANOVA Table (Proc Mixed in SAS) for main effects of treatment, month, commodity, and all associated interactions (*p* < 0.001 for all) for percentage of larvae *Trogoderma granarium* remaining in that stage exposure.

Factors	*F*-Value	df	*p*-Value
Treatment	59.7	2, 178	<0.001
Month	91.3	4, 178	<0.001
Commodity	178.2	2, 178	<0.001
Treatment × Month	2.8	8, 178	0.006
Treatment × Commodity	58.4	4, 178	<0.001
Month × Commodity	9.3	8, 178	<0.001
Month × Treatment × Commodity	2.0	16, 178	0.016

**Table 2:** Percentage of larvae (mean ± SE) of *Trogoderma granarium* that remained in the larval stage on corn, brown rice, and wheat, and each commodity treated with either DiaconIGR^®^ *Plus* or Gravista^®^ formulation at each specific trial month or untreated grain. Bioassays conducted one day after treatment (month 0) and every three months thereafter for one year. Means for columns within month for each treatment are noted with different lower-case letters indicating significant differences among commodities and means within row for each commodity are noted with different capital letters denoting significant differences among treatment within commodity (*p* < 0.05, Proc Mixed, LSMeans with Tukey Adjustment).

Trial Month	Commodity	Controls	DiaconIGR^®^ Plus	Gravista^®^
0	Corn	13.4 ± 5.8aB	85.8 ± 6.1aA	75.7 ± 2.7aA
	Wheat	8.3 ± 2.3aB	24.0 ± 3.6bA	29.0 ± 3.7bA
	Brown Rice	5.1 ± 1.6aB	11.0 ± 0.8cA	3.1 ± 1.1cA
3	Corn	7.3 ± 2.4bB	75.1 ± 6.5aA	59.8 ± 8.5aA
	Wheat	25.0 ± 4.4aB	52.8 ± 9.2aA	43.5 ± 7.4aAB
	Brown Rice	13.4 ± 4.6abA	12.8 ± 3.7bA	10.9 ± 5.0bA
6	Corn	60.7 ± 6.4aB	92.9 ± 2.4aA	85.1 ± 1.9aA
	Wheat	66.3 ± 10.6aA	83.4 ± 6.4aA	79.1 ± 4.1aA
	Brown Rice	61.2 ± 7.9aA	40.9 ± 7.8bA	51.7 ± 6.3bA
9	Corn	24.1 ± 9.4aC	89.6 ± 3.6aA	63.7 ± 6.8aB
	Wheat	11.2 ± 4.1aA	15.4 ± 3.8bA	17.6 ± 4.7bA
	Brown Rice	25.0 ± 6.4aA	7.5 ± 2.7bB	16.7 ± 4.9bAB
12	Corn	14.3 ± 5.4aC	77.6 ± 9.4aA	46.8 ± 6.2aB
	Wheat	11.6 ± 1.6aA	14.9 ± 2.0bA	40.1 ± 6.2aA
	Brown Rice	31.5 ± 6.4aA	6.8 ± 2.2bB	7.5 ± 3.3bB

**Table 3:** Three-way ANOVA Table (Proc Mixed in SAS) for main effects of treatment, month, commodity, and all associated interactions (*p* < 0.001 for all) for percentage of normal pupae developing from exposed larvae of *Trogoderma granarium*.

Factor	*F*-Value	df	*p*-Value
Treatment	5.9	2.178	0.006
Month	24.5	4.178	<0.001
Commodity	0.9	2.178	0.396
Treatment × Month	5.8	8.178	<0.001
Treatment × Commodity	1.3	4.178	0.146
Month × Commodity	1.7	8.178	0.293
Month × Treatment × Commodity	2.0	16.178	0.012

**Table 4:** Percentage of normal pupae (mean ± SE) from larvae of *Trogoderma granarium* exposed on corn, brown rice, and wheat, and each commodity treated with either DiaconIGR^®^ *Plus* or Gravista^®^ formulation or untreated. Bioassays conducted one day after treatment (month 0) and every three months thereafter for one year. Means for columns within month for each treatment are noted with different lower-case letters indicating significant differences among commodities and means within row for each commodity are noted with different capital letters denoting significant differences among treatment within commodity (*p* < 0.05, Proc Mixed, LSMeans with Tukey Adjustment).

Trial Month	Commodity	Controls	DiaconIGR^®^ *Plus*	Gravista^®^
0	Corn	0.0 ± 0.0aA	1.2 ± 1.2aA	0.0 ± 0.0bA
	Wheat	0.0 ± 0.0aC	2.1 ± 3.3aB	1.0 ± 1.0aA
	Brown Rice	0.0 ± 0.0aC	2.8 ± 1.9aA	0.0 ± 0.0bB
3	Corn	4.3 ± 1.8bA	0.0 ± 0.0aA	5.3 ± 2.3aA
	Wheat	10.1 ± 2.7abA	5.1 ± 2.2aA	2.1 ± 1.2aA
	Brown Rice	13.2 ± 4.4aA	5.0 ± 3.4aA	0.0 ± 5.0aA
6	Corn	3.3 ± 2.3aA	0.0 ± 0.0aB	0.0 ± 0.0aB
	Wheat	0.0 ± 0.0aA	0.0 ± 0.0aA	0.0 ± 0.0aA
	Brown Rice	1.0 ± 1.0aA	0.0 ± 0.0aA	0.0 ± 0.0aA
9	Corn	0.0 ± 0.0aA	0.0 ± 0.0aA	0.0 ± 0.0aA
	Wheat	0.0 ± 0.0aA	0.0 ± 0.0aA	0.0 ± 0.0aA
	Brown Rice	0.0 ± 0.0aA	0.0 ± 0.0aA	0.0 ± 0.0aA
12	Corn	0.0 ± 0.0aA	0.0 ± 0.0aA	0.0 ± 0.04aA
	Wheat	0.0 ± 0.0aA	1.3 ± 1.3aA	1.0 ± 1.0aA
	Brown Rice	0.0 ± 0.0aA	0.0 ± 0.0aA	0.0 ± 0.0aA

**Table 5:** Three-way ANOVA Table (Proc Mixed in SAS) for main effects of treatment, month, commodity, and all associated interactions for percentage of pupal–adult intermediates developing from exposed larvae of *Trogoderma granarium*.

Factor	*F*-Value	df	*p-*Value
Treatment	164.9	2.178	<0.0001
Month	29.1	4.178	<0.0001
Commodity	94.8	2.178	<0.0001
Treatment × Month	8.4	8.178	<0.0001
Treatment × Commodity	28.2	4.178	<0.0001
Month × Commodity	6.7	8.178	<0.0001
Month × Treatment × Commodity	3.6	16.178	<0.0001

**Table 6:** Percentage of deformed pupal–adult intermediates (mean ± SE) from larvae of *Trogoderma granarium* exposed on corn, brown rice, and wheat, and each commodity treated with either DiaconIGR^®^ *Plus* or Gravista formulation or untreated. Bioassays conducted one day after treatment (month 0) and every three months thereafter for one year. Means for columns within month for each treatment are noted with different lower-case letters indicating significant differences among commodities and means within row for each commodity are noted with different capital letters denoting significant differences among treatments within commodity (*p* < 0.05, Proc Mixed, LSMeans with Tukey Adjustment).

Trial Month	Commodity	Controls	DiaconIGR^®^ *Plus*	Gravista^®^
0	Corn	0.0 ± 0.0aC	11.0 ± 4.9cB	22.0 ± 3.6bA
	Wheat	0.0 ± 0.0aC	72.8 ± 3.3aA	62.8 ± 3.2aB
	Brown Rice	0.0 ± 0.0aC	39.9 ± 5.7bA	17.8 ± 5.8bB
3	Corn	0.0 ± 0.0aB	24.8 ± 6.5aA	24.4 ± 4.2bA
	Wheat	0.0 ± 0.0aB	41.0 ± 9.6bA	53.4 ± 6.6aA
	Brown Rice	0.0 ± 0.0aB	27.8 ± 6.2aA	11.8 ± 5.0bB
6	Corn	0.0 ± 0.0aB	4.1 ± 1.8aAB	9.6 ± 2.6abAB
	Wheat	0.0 ± 0.0aB	12.7 ± 6.2bA	16.1 ± 4.8aA
	Brown Rice	0.0 ± 0.0aB	16.3 ± 1.1aA	5.9 ± 1.8bAB
9	Corn	1.2 ±1.2aA	8.0 ± 3.4bA	5.5 ± 3.4bA
	Wheat	0.0 ± 0.0aB	46.4 ± 3.3aA	30.9 ± 4.6aA
	Brown	0.0 ± 0.0aB	10.3 ± 4.3bA	2.0 ± 1.3bAB
12	Corn	0.0 ± 0.0aB	13.9 ± 6.3bA	12.1 ± 5.4abA
	Wheat	0.0 ± 0.0aC	47.3 ± 8.6aA	22.2 ± 2.7aB
	Brown Rice	1.2 ± 1.2aB	8.7 ± 5.1bA	4.2 ± 2.0baAB

**Table 7:** Three-way ANOVA Table (Proc Mixed in SAS) for main effects of treatment, month, commodity, and all associated interactions (*p* < 0.001 for all) for percentage of normal adults developing from exposed larvae of *Trogoderma granarium*.

Factor	*F*-Value	df	*p-*Value
Treatment	268.8	2.178	<0.0001
Month	68.8	4.178	<0.0001
Commodity	207.9	2.178	<0.0001
Treatment × Month	14.0	8.178	<0.0001
Treatment × Commodity	67.1	4.178	<0.0001
Month × Commodity	5.6	8.178	<0.0001
Month × Treatment × Commodity	2.9	16.178	<0.0001

**Table 8:** Percentage of normal adults (mean ± SE) from larvae of *Trogoderma granarium* exposed on corn, brown rice, and wheat, and each commodity treated with either DiaconIGR^®^ *Plus* or Gravista formulation or untreated. Bioassays conducted one day after treatment (month 0) and every three months thereafter for one year. Means for columns within month for each treatment are noted with different lower-case letters indicating significant differences among commodities and means within row for each commodity are noted with different capital letters denoting significant differences among treatment within commodity (*p* < 0.05, Proc Mixed, LSMeans with Tukey Adjustment).

Trail Month	Commodity	Controls	DiaconIGR^®^ *Plus*	Gravista^®^
0	Corn	86.6 ± 5.8aA	2.4 ± 1.5bB	2.5 ± 2.5bB
	Wheat	91.7 ± 2.2aA	1.1 ± 1.1bC	6.4 ± 0.9bB
	Brown Rice	94.9 ± 1.5aA	46.2 ± 2.8aC	79.1 ± 5.3 aB
3	Corn	88.4 ± 2.7aA	0.0 ± 0.0bC	10.4 ± 3.2B
	Wheat	65.0 ± 4.7bA	1.0 ± 1.0bB	1.0 ± 1.0B
	Brown Rice	73.5 ± 3.4bA	54.4 ± 8.9aB	77.3 ± 3.5A
6	Corn	33.9 ± 7.1aA	3.0 ± 1.2bB	5.4 ± 4.1bB
	Wheat	33.8 ±10.6aA	3.9 ± 1.7bB	4.1 ± 2.5bB
	Brown Rice	37.7 ± 8.1aA	42.8 ± 12.1aA	42.4 ± 6.2aA
9	Corn	74.8 ±10.2aA	1.2 ± 1.2cB	2.4 ± 1.5cB
	Wheat	88.8 ± 4.1aA	38.2 ± 4.7bB	51.5 ± 6.2bB
	Brown Rice	76.0 ± 6.4aA	82.2 ± 3.9aA	81.2 ± 5.2aA
12	Corn	85.7 ± 5.4aA	8.4 ± 4.9cC	41.1 ± 3.1bB
	Wheat	88.3 ± 1.6aA	36.8 ± 6.9bB	36.7 ± 5.5bB
	Brown Rice	67.3 ± 6.7bA	84.5 ± 5.6aA	88.2 ± 5.2aA

**Table 9:** ANOVA Table (Proc Mixed in SAS) for main effects of treatment, month, commodity, and all associated interactions (*p* < 0.001 for all) for developmental index (range from 20, all larvae, to 80, all normal emerged adults) of *Trogoderma granarium* exposed in the larval stage.

Factor	*F*-Value	df	*p*-Value
Treatment	101.3	2.176	<0.001
Month	64.0	4.176	<0.001
Commodity	146.5	2.176	<0.001
Treatment × Month	4.1	8.176	<0.001
Treatment × Commodity	47.3	4.176	<0.001
Month × Commodity	6.1	8.176	<0.001
Month × Treatment × Commodity	1.5	16.176	0.10

**Table 10:** Index values (mean ± SE) for development of *Trogoderma granarium* larvae after exposure on corn, brown rice, and wheat, and each commodity treated with either DiaconIGR^®^ *Plus* or Gravista^®^ formulation. Bioassays conducted one day after treatment (month 0) and every three months thereafter for one year. Index values range from 20 (complete arrestation of larvae) to 80 (all exposed larvae reached the adult stage). Means for columns within month for each treatment are noted with different lower-case letters indicating significant differences among commodities and means within row for each commodity are noted with different capital letters denoting significant differences among treatment within commodity (*p* < 0.05, Proc Mixed, LSMeans with Tukey Adjustment).

Trail Month	Commodity	Controls	DiaconIGR^®^ *Plus*	Gravista^®^
0	Corn	72.0 ± 3.4aA	26.1 ± 2.5cB	30.2 ± 1.2cB
	Wheat	72.0 ± 1.3aA	50.2 ± 1.4bAB	42.2 ± 1.2bB
	Brown Rice	76.9 ± 0.9aA	64.3 ± 1.9aA	74.6 ± 1.0aA
3	Corn	73.9 ± 1.4aA	29.9 ± 2.6bB	37.1 ± 3.6bB
	Wheat	61.0 ± 2.7abA	38.2 ± 3.8bB	42.4 ± 3.0bB
	Brown Rice	66.7 ± 1.9abA	64.7 ± 1.9aA	64.8 ± 3.0aA
6	Corn	42.2 ± 4.1aA	23.5 ± 1.4bB	27.0 ± 1.5bB
	Wheat	40.3 ± 6.3aA	27.4 ± 2.7bA	29.2 ± 1.7bA
	Brown Rice	42.8 ± 4.8aA	52.2 ± 5.4aA	47.8 ± 3.7aA
9	Corn	65.3 ± 5.8aA	24.6 ± 1.6bB	40.6 ± 3.8bAB
	Wheat	61.5 ± 2.3aA	63.4 ± 2.9aA	63.3 ± 2.8aA
	Brown Rice	65.0 ± 3.8aA	73.3 ± 1.4aA	69.6 ± 3.0aA
12	Corn	71.4 ± 3.2aA	30.6 ± 4.5cB	49.4 ± 3.9bB
	Wheat	73.0 ± 1.0aA	48.9 ± 12.2bA	51.1 ± 3.42bA
	Brown Rice	60.9 ± 3.9aA	74.2 ± 1.7aA	74.6 ± 1.9aA

## Data Availability

Please request any particular data through the corresponding author. Public data will be added to the USDA NAL AG commons and data repository.
